# Supplementary material for: Scenic spot path planning and journey customization based on multilayer hybrid hypernetwork optimization
Source: PLoS One. 2024 Dec 10;19(12):e0308135. doi: 10.1371/journal.pone.0308135 (PMC11630575; doi:10.1371/journal.pone.0308135)
Supplement: Attachment — Submitted filename: Response.docx [file pone.0308135.s002.docx]

PONE-D-24-07129R1

**Comments to the Author**

6. Review Comments to the Author

Please use the space provided to explain your answers to the questions above. You may also include additional comments for the author, including concerns about dual publication, research ethics, or publication ethics. (Please upload your review as an attachment if it exceeds 20,000 characters)

Reviewer #1: To realize the efficient matching between tourists and scenic spots, this paper proposes (1) the feature extraction method of adaptive multi-path selection (AMS), (2) the tourist personalized reasoning method based on multi-layer hybrid network (MHN), and (3) the path planning method based on hyper-network optimization (HO).

However, there are still several aspects need be improved.

(1) The notations are chaotic and their connections are weak.

Response: I modify the notations as follows:

$$s_{j}=S[s_{i}]$$

(3)

$$f_{k}(x_{k})=\min_{p_{kn}\in P (x_{k})}W(x_{k},p_{kn})$$

(5)

(2) The optimization objective function (see, Eq. (5)) is uninformative and very difficult to understand.

Response:I add the content to understand the optimization objective function, as follows:

When considering how to plan a tour route to maximize tourist satisfaction, equation 5 can serve as a crucial mathematical modeling tool to guide our decision-making. In the tourism industry, the design of a route that maximizes tourist satisfaction takes into account factors such as transportation, accommodation, catering, attractions, and tourists' time.

(3) The accuracy of expression urgently needs to be improved. For example, the ``OH'' listed in Table 2 should be ``HO''.

Response: I correct the mistake in Table 2, as follows:

**Table 2 Ablation experiments**

| AMS | MHN | HO | mAP | Acc | L | T (ms) |
| --- | --- | --- | --- | --- | --- | --- |
|  |  |  | 0.775 | 0.782 | 22 | 234 |
| O |  |  | 0.811 | 0.804 | 18 | 192 |
|  | O |  | 0.804 | 0.811 | 17 | 189 |
|  |  | O | 0.816 | 0.807 | 19 | 199 |
| O | O |  | 0.834 | 0.838 | 15 | 201 |
| O |  | O | 0.842 | 0.842 | 14 | 215 |
|  | O | O | 0.839 | 0.833 | 15 | 228 |
| O | O | O | 0.881 | 0.877 | 12 | 236 |

(4) The best result should be highlighted, such as in bold.

Response: I highlight the best results in tables, as follows:

**Table 3 Comparison AMS with other methods**

| Methods | mAP | mAP50 | mAP75 | mAP95 |
| --- | --- | --- | --- | --- |
| CNN-FPN | 0.753 | 0.789 | 0.762 | 0.732 |
| Self-Attention | 0.797 | 0.835 | 0.803 | 0.753 |
| Uniformer | 0.801 | 0.824 | 0.811 | 0.786 |
| LiConvFormer | 0.785 | 0.831 | 0.796 | 0.767 |
| Ours | **0.811** | **0.846** | **0.814** | **0.796** |

**Table 4 Compare KPD with other methods**

| Methods | Acc | GFLOPs | Cost time (ms) |
| --- | --- | --- | --- |
| SCN_GNN | 0.831 | 199 | 110 |
| RT-GCN | 0.829 | 211 | 135 |
| MSA-GCN | 0.815 | 204 | 121 |
| Ours | **0.838** | **193** | **102** |

(5) If the AMS, MHN, and HO are not considered, what exactly is the baseline-model?

Response: I introduce the baseline in Section 4.2, as follows:

We employ the CNN as our baseline. Combining the results of attraction recommendations and geographic information data, CNN can assist in planning optimal travel routes. By analyzing information such as the geographic location, traffic conditions, and tourist flow of attractions, CNN can help tourists select the most suitable tour sequence and transportation mode.

(6) In the 20th page of this PDF file, there is a mistaken code between 0.50 and 0.95.

Response: I correct this mistake, as follows:

We used mAP50, mAP75, mAP95, and mAP to represent the mean average precision (AP) of all classes when the Intersection over Union (IoU) was is equal to 0.50, 0.75, 0.95, and 0.50 : 0.95, respectively.

(7) The expression of first person such as ``We'' or ``I'' should be unified.

Response: I unify the ``We'' or ``I'', as follows:

Finally, we conduct a thorough investigation into the performance of the hyper-network optimization-based path planning method (HO), and we utilize a nonlinear objective function for all comparisons.

(8) The path planning and path prediction are two completely different problems.

Response:

Response: I add the introduction of path planning as follows:

Tourism route planning research involves multiple aspects, including understanding the destination, developing the itinerary theme, designing diversified travel routes, arranging a reasonable schedule, selecting transportation modes, arranging accommodations, planning meals, and organizing activities.
